# Evaluating Tuberculosis Surveillance Using Global Standards and Benchmarks in the Philippines: Mixed Methods Study

**DOI:** 10.2196/77058

**Published:** 2026-04-06

**Authors:** Minal Ahson, Donna Mae Gaviola, Stephanie O'Connor, Andro Gutierrez, Marvin Apas, Maria Alezandra Eguia, Devon Ray Pacial, Arianne Zamora, Romel Lacson, Alethea De Guzman, Patrick K Moonan

**Affiliations:** 1 Division of Global HIV and Tuberculosis, Centers for Disease Control and Prevention, 1600 Clifton Road NE, Atlanta, GA, 30333, United States, 1 4044018024; 2 Epidemic Intelligence Service, Office of Workplace Development, Centers for Disease Control and Prevention, Atlanta, GA, United States; 3 Department of Health, Epidemiology Bureau, Manila, Philippines; 4 Department of Health, Knowledge Management and Information Technology Service, Manila, Philippines; 5 Division of Global HIV and Tuberculosis, Centers for Disease Control and Prevention, Manila, Philippines

**Keywords:** surveillance, tuberculosis, tuberculosis surveillance, disease surveillance, public health surveillance, data quality, case notification, electronic health records, health information systems, Philippines, surveillance evaluation

## Abstract

**Background:**

The Philippines accounts for 7% of the global tuberculosis (TB) burden. In 2022, an estimated 741,000 Filipinos developed TB, and 40,000 died as a result. Approximately 350,000 new TB diagnoses remain unreported annually.

**Objective:**

In October 2023, we undertook an assessment of the accuracy, completeness, and timeliness of the national TB surveillance system using globally accepted TB surveillance standards and benchmarks.

**Methods:**

A team of Filipino and international evaluators conducted field visits to 9 purposively selected health facilities across 4 regions. Health records were reviewed, and key informants were interviewed to assess essential activities and best practices for TB surveillance.

**Results:**

Of 16 applicable performance standards, 4 (25%) were fully met, 9 (56.3%) were partially met, and 3 (18.8%) were not met. This was an improvement from a similar assessment conducted in 2019. Although electronic case-based recording and reporting had expanded greatly since 2019, large local variations in the adoption of quality assurance practices were evident. More than 50% of persons with reported TB had no bacteriological confirmation despite the presence of a functional national laboratory network. Underreporting due to underdiagnosis was also noted. Delayed reporting was common, especially in locations with limited internet connectivity or limited access to anti-TB medications.

**Conclusions:**

Issues related to data quality assurance, gaps in case verification, and timeliness emerged as potential threats to data integrity and surveillance fidelity. Enhanced monitoring and evaluation, along with tailored studies, such as a national TB inventory study, could assist in quantifying potential underreporting and clinical overdiagnosis, guiding future funding, and assessing progress toward elimination targets.

## Introduction

Surveillance is a key component of global efforts to eliminate tuberculosis (TB) [1,2]. To support countries in strengthening their surveillance systems and achieving TB management goals, the World Health Organization (WHO) developed a standardized assessment tool that countries can use to evaluate the performance of their TB surveillance and vital registration systems [3].

The Philippines currently ranks fourth in the list of countries with the highest TB incidence and accounts for 7% of the global TB burden [4]. WHO incidence estimates for 2022 indicated approximately 741,000 incident TB cases in the Philippines compared with 435,890 notified new and retreatment cases, yielding a treatment coverage of 59% [5]. The estimated number of unreported TB cases reflects the residual between WHO-modeled incidence and observed notifications (derived from the WHO’s standardized incidence estimation framework rather than from calculations performed by the authors) [5]. The Philippines Department of Health introduced an internet-based TB surveillance system, the Integrated TB Information System (ITIS), in 2016 [6]. This facilitates the systematic recording and reporting of case-based information in accordance with National Tuberculosis Control Program (NTP) guidance [7,8]. Currently, TB case data are captured from all primary public facilities and several tertiary facilities. A growing number of private healthcare practitioners also participate in mandatory TB notification [9]. All participating network facilities collect TB information in logs and then use ITIS to enter data electronically. Quality assurance procedures guide systematic data entry. Data are reviewed and validated by provincial and regional NTP offices each quarter. The national NTP office reviews data annually and produces an annual TB report to document programmatic performance over time [10].

We aimed to assess the ability of the NTP to accurately enumerate TB cases and deaths using the WHO’s TB surveillance assessment tool, which comprises a checklist of standards and benchmarks covering metrics such as TB data quality, surveillance system coverage, and treatment coverage [11]. The previous surveillance evaluation conducted in 2019 using the same standards and benchmarksfound that a total of 5 benchmarks were met, 1 was partially met, 6 were not met, and 5 were not applicable (Multimedia Appendix 1).

## Methods

### Overview

In October 2023, a team of Filipino and international evaluators from the Epidemiology Bureau of the Philippines Department of Health and the US Centers for Disease Control and Prevention (CDC) conducted field visits to 9 health facilities in 4 of the country’s 18 regions: Calabarzon, Cordillera Administrative Region, Ilocos Region, and National Capital Region. The facilities were selected to gain perspectives from a variety of settings and included urban and rural, primary and tertiary care, and private and government settings. These included regional and provincial NTP offices. We interviewed stakeholders (including clinicians, program managers, and frontline workers) to learn about essential activities and best practices regarding TB surveillance. Each visit included a presentation from the facility, discussion of surveillance processes, and a walkthrough of TB-specific areas. Selected paper-based registries, clinical records, and electronic-based data (ITIS) were reviewed for accuracy, completeness, and timeliness. Additional publicly available data sources were also consulted [12-14]. After site visits and discussion with local and national partners, the WHO’s assessment tool checklist was completed and compared with the previous assessment conducted in 2019 [11,12]. The previous TB surveillance assessment used the same WHO standards and benchmarks [12]. That assessment included field visits to a comparable number and mix of health facilities across selected regions of the Philippines, representing varied geographic, administrative, and health system contexts. While the specific facilities and regions assessed in 2019 were not identical to those included in the 2023 assessment, both evaluations were designed to capture perspectives from urban and rural settings and from multiple levels of the health system, allowing for broad comparability of findings over time. This evaluation focused on 5 standards: TB surveillance system data quality, system coverage and vital registration, specific populations, TB treatment outcomes, and programmatic management of TB preventive treatment. We used the same set of 16 standards and benchmarks as in the previous assessment; changes between successive versions of the checklist were relatively minor. An indicator was considered “partially met” if at least one, but not all, of the outcome measures for each benchmark was met.

### Ethical Considerations

This activity was reviewed by the CDC and deemed not research and was conducted consistent with applicable federal law and CDC policy (see, eg, Title 45, part 46.102(l)(2), and Title 21, part 56, of the Code of Federal Regulations and Title 42, §241(d); Title 5, §552a; and Title 44, §3501 et seq of the United States Code [15]). As this activity was classified as a public health surveillance evaluation rather than human subjects research, formal written informed consent was not required. Key informants were verbally informed of the voluntary nature of their participation prior to being interviewed. No personally identifiable patient information was collected or retained. Participants received no compensation.

## Results

On the basis of 2022 data, of the 17 total checklist indicators, of which 1 (5.9%) was not applicable, 4 (23.5%) were fully met, 9 (52.9%) were partially met, and 3 (17.6%) were not met (Multimedia Appendix 1).

### TB Surveillance System Data Quality

Benchmarks B1.1 to 1.3 (B1.4 was not applicable to electronic systems), which relate to case-level data capture, were fully met (Multimedia Appendix 1). Case definitions were consistent with WHO guidelines and the TB case-based reporting framework (B1.1) [2,3]. Persons with TB were identified using unique, anonymized registration numbers. The surveillance system captured a minimum set of variables for all cases (B1.2). These included sociodemographic characteristics (ie, age, sex, and residential address), date of registration, clinical and diagnostic information (ie, anatomical site of disease, radiographic interpretations, and treatment history), laboratory test results, and current treatment regimens. Test results and treatment status were recorded as patients moved through the care pathway. Information was captured in near real time; once data was entered at local levels, they were immediately available for view by the higher-level offices. We observed that ITIS was widely used and that sufficient training and infrastructure were in place. This included training on data collection and entry into the electronic system. Infrastructure included computer availability, electricity, and internet connection at various facilities. However, several users reported that, on occasion, ITIS was slow to load and unsaved data could be lost due to unstable internet connectivity. In facilities with insufficient internet bandwidth, paper-based registers were transferred for entry at hubs with adequate connectivity. In addition, ITIS was not fully integrated with laboratory information systems. Laboratory staff manually entered results into ITIS; automated reporting of molecular-based testing (ie, Xpert MTB/RIF; Cepheid) was not in place.

The B1.5 benchmark, which measures the accuracy, completeness, and internal consistency of data in the national database, was partially met. ITIS has integrated mechanisms to ensure data quality, such as checking for duplicate names with the same spelling. However, although less than 1% of records were invalid or incomplete (judged against a minimal set of variables, including sociodemographic characteristics, date of registration, and clinical and diagnostic variables), the internal mechanisms did not include checking for duplicate entries with different identifiers or spelling, and no evidence of addressing these potential duplications was observed. Furthermore, data validation checks were inconsistent and widely varying. ITIS features a simple, one-click validation step, which users were able to demonstrate during site visits. While ease of use can be an advantage, the current validation process may be insufficient to verify the accuracy of the entered data. Because HIV-associated information is restricted at some levels (ie, patient records are confidential and not integrated into ITIS), validation of records for people with HIV-TB appeared limited. Electronic surveillance systems provide opportunities to automate a portion of the data quality assurance process, but only minimal automated checks for internal validity were in place.

The B1.6 and B1.7 benchmarks related to the internal and external consistency of TB surveillance data, respectively, were also only partially met. Among newly notified persons with TB in 2022, a total of 8% were children aged 0 to 14 years. This was within the recommended acceptable range standard (5%‐15% for low- and middle-income countries). Across all ages, only 50% of reported persons with TB had bacteriological confirmation, well below the 70% to 90% recommended standard [4]. Surveillance data appeared to be internally consistent over time even after considering year-to-year differences experienced during the COVID-19 pandemic. The ratio of pulmonary to extrapulmonary TB was 37:8, and for male to female patients, it was 26:14, which was consistent with pre–COVID-19 levels. The proportion of newly notified TB cases had steadily increased to 49.6% (Table 1; [13,14,16]).

**Table 1.** Selected tuberculosis (TB) surveillance system indicators (the Philippines, 2018‐2022)^a^.

	2018	2019	2020	2021	2022
TB programmatic indicator					
Ratio of notified pulmonary to extrapulmonary TB cases	32:7	36:7	22:5	28:6	37:8
Ratio of male TB cases to female TB cases	21:11	24:13	2:1	2:1	26:14
Childhood TB cases out of all TB cases (%)	13.9	11.6	7.7	7.0	8.1
Proportion of newly notified TB cases with bacteriological confirmation (%)	36.1	33.9	43.9	45.0	49.6
Year-to-year change in new case notification rate (%)	8.4	9.2	–59.5	20.2	26.2
Vital registration indicator					
Proportion of documented primary cause of death among all registered deaths (%)	96.3	96.3	95.7	93.5	94.2
Deaths attributed to TB—all forms, n (%)	23,409 (4.0)	23,877 (3.8)	19,430 (3.2)	19,952 (2.3)	20,588 (3.0)

a As a population-level health system indicator, the universal health coverage service index was 48 in 2010, 57 in 2015, 60 in 2017, 60 in 2019, and 58 in 2021 [13,14,16].

### System Coverage and Vital Registration

B1.8 assesses how well the surveillance system captures TB cases. By default, ITIS only captures persons who received treatment at NTP facilities. Despite mandating notifications from all private health care providers who diagnose persons with TB in 2018, only selected health care providers complied, leading to occasional nonreporting among private sector facilities. Without a complete census or representative survey of private facilities, it is unknown how many facilities are not reporting in ITIS.

In a few facilities, staff were uncertain of who was responsible for reporting TB cases and validating records; in others, staff asserted that they were only responsible for entering data into ITIS for TB treatment initiations, not TB diagnoses. Both scenarios may result in underreporting. A few facilities reported waiting to encode diagnosed patients until treatment was initiated. This practice had major implications for reporting timeliness in settings where supply chain issues have caused months-long treatment initiation delays. Facilities without the human resource capacity to enter data into ITIS on-site or with limited internet connectivity sent TB registers to regional or provincial offices for encoding, resulting in reporting and patient referral delays. In light of such evidence, the B1.8 benchmark was deemed as only partially met.

Although access to health care in the Philippines has steadily improved over the past decade (Table 1), the universal health coverage index was 58 in 2021, well below the recommended benchmark of 80. Thus, the B1.9 benchmark was not met (Multimedia Appendix 1). However, benchmark B1.10, which relates to the coverage and quality of vital registration data provided by the civil registration and vital statistics system, was partially met. According to the Philippine Statistics Authority, the primary cause of death was documented for 640,175 (94.2%) of 679,766 registered deaths in 2022, including 20,588 (3%) attributed to all forms of TB (Table 1). However, the extent of mortality reporting and recording was variable between regions.

### Specific Populations

Of the 3 supplementary indicators pertaining to specific TB populations (people with drug-resistant TB, people with HIV coinfection, and children), only the B2.1 benchmark was met. In 2022, 52% of new cases were diagnosed without laboratory confirmation despite increased Xpert MTB/RIF testing. However, among patients with bacteriologically confirmed pulmonary TB, the vast majority (97%) had drug susceptibility test results, and a total of 9791 rifampin-resistant cases were detected. This meant that benchmark B2.1 was met.

Benchmark B2.2 was not met because, in 2022, the proportion of patients with newly reported TB tested for HIV (70%) remained below the recommended target of 80%. The data in Table 2 show that not only has the annual proportion of patients with newly notified TB tested for HIV increased in recent years but the proportion of patients with TB who tested positive for HIV has also remained low (range 0.8%‐1.5%). Moreover, the proportion of persons living with HIV who initiated antiretroviral treatment after testing positive in the last 3 years was exceptional; only 1 person with newly discovered TB and HIV coinfection did not start antiretroviral treatment.

**Table 2.** HIV testing and percentage of positivity among persons with newly reported tuberculosis (TB; the Philippines, 2018‐2022)^a^.

Indicator	2018	2019	2020	2021	2022
Persons with TB with a documented HIV test result, n (% of total)	101,681 (31)	136,749 (38)	85,672 (39)	109,952 (39)	263,846 (70)
Persons with TB who tested positive, n (% of persons tested)	1477 (1.5)	1615 (1.2)	898 (1.0)	1350 (1.2)	2201 (0.8)
Persons with TB who tested positive and initiated ART^b^, n (% of positive cases)	1350 (91)	1509 (93)	898 (100)	1349 (99)	2201 (100)

a Source: Global TB Data Repository [14] .

b ART: antiretroviral therapy.

In 2022, children and adolescents aged 0 to 14 years accounted for 8% of all reported persons with TB (Table 1). Benchmark B2.3 requires a rate ratio of cases notified among children aged 0 to 4 years to those aged 5 to 14 years in the range of 1.5 to 3.0; the observed ratio of 0.82 fell outside the expected range, and therefore benchmark B2.3 was not met. A ratio lower than expected suggests that TB cases are proportionally higher among older children, which may reflect either underdiagnosis in children below the age of 5 years, overdiagnosis in children and adolescents aged 5 to 14 years, or both. Formal investigations of childhood TB epidemiology to determine the extent of under- or overreporting have not been conducted.

### TB Treatment Outcomes

Both treatment outcome standards (B3.1 and B3.2) were partially met. We observed variations in the accuracy, completeness, and consistency of TB treatment recording and reporting by both setting and region. However, individual treatment outcomes were disaggregated by treatment history, HIV status, and drug resistance test results. Overall, there was limited evidence of local-level surveillance data use. Facilities generally focused on variables associated with targets and on measuring success based primarily on target achievement. We found no evidence that checks had been made to verify that the number of patients with an assigned treatment outcome in any given year matched the number of patients notified the preceding year.

### Programmatic Management of TB Preventive Treatment

While monitoring and evaluation indicators for TB preventive therapy (TPT) were consistent with global guidance [17] and the data collected included the minimum set of variables (people living with HIV, children aged <5 years, individuals aged ≥5 years, and TPT completion), monitoring of TPT was noted in a few settings, and there was significant variation. Thus, B4.2 (TPT programmatic management data were accurate, complete, and consistent) was only partially met (Multimedia Appendix 1).

## Discussion

### Principal Findings

The global standards and benchmarks for TB surveillance and vital registration systems were developed to be applicable to TB control programs in various contexts [3]. By implementing this tool, we demonstrated that it is both applicable and feasible in the Filipino context. Overall, this assessment was an improvement from the previous assessment in 2019, in which 5 benchmarks were met, 1 was partially met, 6 were not met, and 5 were not applicable [12]. In this assessment, fewer indicators were not met, and more were fully or partially assessed.

This assessment revealed progress in several key areas of TB surveillance since the last review. Most notable was the impressive expansion and implementation of electronic case-based recording and reporting. However, we also identified several opportunities for further improvement.

### Improving Data Quality

Data quality issues predominantly stemmed from staff time constraints and a lack of coordination between facilities. Procedures were in place to encourage data quality assurance prior to reporting. However, greater use of automated checks for internal validity, data completeness, and deduplication could reduce the burden on data entry staff and the risk of human error in the reporting process. Losses to follow-up and the number of duplicate records could be reduced by implementing monitoring at higher administrative levels of patients who are transferred but unclaimed in ITIS.

It should be noted that, while the proportion of bacteriologically confirmed pulmonary TB with drug susceptibility test results was considerably higher than the 80% recommended standard (Multimedia Appendix 1), the proportion of all TB cases with bacteriological confirmation still ranked among the lowest in the world [5]. Proposed explanations for the low rates of bacteriological confirmation include health care provider mistrust of molecular testing results, underuse of molecular diagnostics, and the high number of specimens rejected by laboratories due to low-quality sputum collection.

### Hastening the Transition From Aggregated Paper-Based to Electronic Case-Based Recording and Reporting

ITIS represents a triumph in the transition to electronic, person-level reporting. However, it is hampered by slow loading speeds, which prevents users from accessing the system. Taking steps to reduce loading times, such as increasing the number of servers, would significantly improve both user satisfaction and the timeliness of data entry and is highly recommended. A mobile-based platform, which does not require a continuous internet connection or expensive hardware, would also help overcome connectivity barriers. Initiating automated transmission of molecular test results could also improve efficiency and data quality. Shifting from manual to automated validation measures offers the dual benefit of reducing the burden on staff and improving data quality. Increasing the number of dedicated personnel at each health facility would expedite implementation of these changes and ensure continuity.

### Reducing Underreporting and Underdiagnosis

Ensuring that all persons with TB have timely access to treatment will help reduce household contact transmission risk and case reporting delays; resolving supply chain issues will further support this. Increasing the proportion of people with bacteriologically confirmed TB would also help tackle the problem of underreporting and underdiagnosis; while clinical diagnoses are important in the absence of laboratory-based alternatives, they can result in under- or misdiagnosis, especially for drug-resistant TB. Pooling samples and providing standardized sample collection instructions to facilities could improve bacteriological diagnosis and reduce the number of rejected sputum samples. Thus, studies to determine the causes of overreliance on clinical diagnosis and the extent of underreporting in the private sector are strongly recommended.

### Promoting the Use of Surveillance Data to Drive Public Health Decision-Making

Informing public health action is a central objective of conducting disease surveillance activities. Training public health workers to analyze and use their own data could be undertaken in collaboration with epidemiology and surveillance units as well as the NTP.

### Limitations

This evaluation took place over a short period and included a small number of field observations at 9 representative facilities. This limitation is particularly important given the geographic and socioeconomic heterogeneity of the Philippines. In addition, we focused on direct observation in reporting facilities and interviews with key informants rather than the more in-depth data evaluation that traditionally takes place during a larger and more formal epidemiologic or TB program review.

### Conclusions

Data quality assurance, case verification, and timeliness were identified as potential risks to data integrity and TB surveillance fidelity in the Philippines. Enhanced monitoring and evaluation, along with tailored studies, such as a national TB inventory study, are proposed as possible measures to quantify underreporting and clinical overdiagnosis, guide future funding, and assess progress toward TB elimination targets.

## Supplementary material

10.2196/77058 Multimedia Appendix 1 Benchmarks for tuberculosis surveillance in the Philippines (2019 and 2023).

## Abbreviations

CDC: Centers for Disease Control and Prevention
ITIS: Integrated Tuberculosis Information System
NTP: National Tuberculosis Control Program
TB: tuberculosis
TPT: tuberculosis preventive therapy
WHO: World Health Organization
