# Supplementary material for: Evaluating Tuberculosis Surveillance Using Global Standards and Benchmarks in the Philippines: Mixed Methods Study
Source: JMIR Public Health Surveill. 2026 Apr 6;12:e77058. doi: 10.2196/77058 (PMC13054790; doi:10.2196/77058)
Supplement: Multimedia Appendix 1 [file publichealth-v12-e77058-s001.docx]

**Table S1. Benchmarks for TB surveillance in the Philippines, 2019 and 2023**

| **Standard** | | **Benchmarks** | | **2019** | **2023** |
| --- | --- | --- | --- | --- | --- |
| **Section 1 – Core standards** | | | | | |
| **TB surveillance system data quality** | | | | | |
| *B1.1 Case definitions are consistent with WHO guidelines* | | All three benchmarks should be satisfied to meet this standard:   - Laboratory-confirmed cases^[[1]](#endnote-1)^ are distinguished from clinically diagnosed cases - New cases are distinguished from previously treated cases - Pulmonary cases are distinguished from extrapulmonary cases | | Met  Partially met  Not met | Met  Partially met  Not met |
| *B1.2 The TB surveillance system is designed to capture a minimum set of variables for reported TB cases* | | Data are routinely collected for each of the following variables, as a minimum:   - Age or age group - Sex - Year of registration - Bacteriological results - History of previous treatment - Anatomical site of disease - For case-based systems, a patient identifier | | Met  Partially met  Not met | Met  Partially met  Not met |
|  | *B1.3 All scheduled periodic data submissions have been received and processed at the national level* | | *For paper-based systems:*   - 100% of expected reports from each TB basic management unit have been received and data have been aggregated at national level   *For national patient-based or case-based electronic systems that import data files from subnational (e.g. provincial or regional) electronic systems:*   - 100% of expected data files have been imported | Met  Partially met  Not met  Not applicable | Met  Partially met  Not met  Not applicable |
|  | *B1.4 Data in quarterly reports (or equivalent) are accurate, complete, and internally consistent (for paper-based systems only)* | | All benchmarks should be satisfied to meet this standard:   - Subtotals of the number of TB cases by age group, sex and case type equal the total number of reported TB cases in ≥95% of quarterly reports (or equivalent) from BMUs - The number of TB cases in ≥95% of quarterly reports (or equivalent) matches the number of cases recorded in BMU TB registers and source documents (patient treatment cards and laboratory register) - Data for a minimum set of variables are available for ≥95% of the total number of reported TB cases in quarterly reports | Met  Partially met  Not met  Not applicable | Met  Partially met  Not met  Not applicable |
|  | *B1.5 Data in national database are accurate, complete, internally consistent, and free of duplicates (For electronic case-based or patient-based systems only)* | | All benchmarks should be met to reach this standard:   - Data validation checks are in place at national level to identify and correct invalid, inconsistent, and missing data in the minimum set of variables (B1.2) - For each variable in the minimum set (standard B1.2), ≥90% of case records are complete, valid and internally consistent for the year being assessed - <1% of case records in the national dataset for the year being assessed are unresolved potential duplicates | Met  Partially met  Not met  Not applicable | Met  Partially met  Not met  Not applicable |
|  | *B1.6 TB surveillance data are externally consistent* | | All benchmarks should be met to reach this standard:   - Percentage of bacteriologically confirmed cases among pulmonary new and relapse cases ranges between 70% and 90% - Year-to-year change of TB notification rates (new relapse, all forms) is consistent with the year-to-year change in bacteriologically confirmed notification rates for pulmonary TB (i.e. the trajectories are in the same direction) - Overall percentage of decline in proportion of bacteriologically confirmed pulmonary TB cases over the 5 years preceding the year of the assessment does not exceed 5% | Met  Partially met  Not met | Met  Partially met  Not met |
|  | *B1.7 Number of reported TB cases is internally consistent* | | If vital registration data *are* available, then the following benchmark should be satisfied for this standard to be met:   - Year-to-year change in the national number of reported TB cases is consistent with year-to-year change in national TB mortality (HIV-negative, from national vital registration; that is, trajectories are in the same direction)   If vital registration data are *not* available, then the following benchmarks should be satisfied for this standard to be met:   - Ratio of notified pulmonary to extrapulmonary TB cases - Ratio of male to female TB cases - Proportion of childhood TB out of all TB cases - Year-to-year change in the case notification rate for all forms of TB - Year-to-year change in the case notification rate for new bacteriologically confirmed TB - Ratio of the number of people with presumptive TB to total notifications of TB cases (if data are available) | Met  Partially met  Not met | Met  Partially met  Not met |
|  | **System coverage** | | | | |
|  | *B1.8 All diagnosed cases of TB are reported* | | All benchmarks should be satisfied to meet this standard:   - TB reporting is a legal requirement - All case types, including drug-resistant TB, are included in the overall number of cases reported - ≥90% of TB cases are reported to national health authorities, as determined by a national-level investigation (e.g. inventory study) conducted within the past 10 years | Met  Partially met  Not met | Met  Partially met  Not met |
|  | *B1.9 Population has good access to health care* | | - UHC index score is ≥80 (SDG Indicator 3.8.1) | Met  Partially met  Not met | Met  Partially met  Not met |
|  | **Vital registration** | | | | |
|  | *B1.10 Vital registration system has high national coverage and is of high quality* | | - Vital registration data provided by CRVS is evaluated as either “1-High” or “2-Medium”   (See *WHO methods and data sources for country-level causes of death 2000–2019*, at [*ghe2019_cod_methods.pdf (who.int)*](https://cdn.who.int/media/docs/default-source/gho-documents/global-health-estimates/ghe2019_cod_methods.pdf?sfvrsn=37bcfacc_5)) | Met  Partially met  Not met | Met  Partially met  Not met |
|  | **Section 2 – Supplementary standards for specific populations** | | | | |
|  | **Standard** | | **Benchmarks** | **2019** | **2023** |
|  | *B2.1 Surveillance data provide a direct measure of rifampicin-resistant TB in bacteriologically confirmed pulmonary cases* | | One of these two benchmarks should be satisfied to meet this standard:   - Rifampicin susceptibility testing results are documented for ≥80% of all bacteriologically confirmed pulmonary TB cases - Rifampicin susceptibility testing results are documented for a nationally representative drug resistance survey conducted in the past 5 years | Met  Partially met  Not met | Met  Partially met  Not met |
|  | *B2.2 Surveillance data provide a direct measure of the prevalence of HIV infection in TB cases* | | One of these two benchmarks should be satisfied to meet this standard:   - HIV status (positive/negative) is documented for ≥80% of all notified TB cases - HIV status is available from a representative sample from all TB cases notified, in settings where there is a low-level epidemic state and it is not feasible to implement routine surveillance | Met  Partially met  Not met | Met  Partially met  Not met |
|  | *B2.3 Surveillance data for children reported with TB (defined as ages 0–14 years) are reliable and accurate AND all diagnosed childhood TB cases are reported* | | Both these benchmarks should be satisfied to meet this standard:   - Rate ratio of groups aged 0–4 to 5–14 years is in the range 1.5–3.0 - ≥90% of childhood TB cases are reported to national health authorities, as determined by a national-level investigation (e.g. inventory study) conducted in the past 10 years | Met  Partially met  Not met | Met  Partially met  Not met |
|  | **Section 3 – Treatment outcomes** | | | | |
|  | **Standard** | | **Benchmarks** | **2019** | **2023** |
|  | *B3.1 Monitoring treatment outcomes is consistent with WHO guidelines* | | Both these benchmarks should be satisfied to meet this standard:   - Treatment outcome definitions for all TB cases are consistent with WHO guidelines - Treatment outcomes of TB patients at national level can be disaggregated by at least the following variables: treatment history, HIV status and drug resistance status | Met  Partially met  Not met  Not assessed | Met  Partially met  Not met |
|  | *B3.2 Recording and reporting of TB treatment outcomes are accurate, complete and consistent* | | All these benchmarks should be satisfied to meet this standard:  *For paper-based systems:*   - Assignment of treatment outcomes is correct for >95% of TB patients recorded in the facility register - Number of treatment outcomes (for each outcome category) in >95% of quarterly reports (or equivalent) matches the number recorded in BMU TB registers - Reported number of the cohort of patients with an expected assigned^a^ treatment outcome in any given year matches the number of patients notified the year before - <1% of cases are assigned an outcome of not evaluated   *For case-based or patient-based digital systems:*   - Data validation checks are in place to ensure validity of assigned treatment outcome for individual cases - Reported number of the cohort of patients with an expected assigned^1^ treatment outcome in any given year matches the number of patients notified the year before - <1% of cases are assigned an outcome of not evaluated | Met  Partially met  Not met  Not assessed | Met  Partially met  Not met |
|  | **Section 4 – Programme management of TB preventive treatment** | | | | |
|  | **Standard** | | **Benchmarks** | **2019** | **2023** |
|  | *B4.1 Monitoring indicators for PMTPT are consistent with WHO guidelines* | | All these benchmarks should be satisfied to meet this standard:   - M&E indicators for PMTPT are consistent with WHO guidelines in terms of:   - Contact investigation coverage   - TPT coverage (disaggregated by PLHIV, contacts <5 years of age and ≥5 years)   - TPT completion (disaggregated by regimens lasting 6 months or more and others lasting <6 months) - PMTPT dataset contains the minimum variables for monitoring TPT at three important instances of PMTPT:   - Assessment of contacts of TB patients   - Assessment of PLHIV and other at-risk groups   - Initiation and completion of TPT | Met  Partially met  Not met  Not assessed | Met  Partially met  Not met |
|  | *B4.2 PMTPT data are accurate, complete and consistent* | | All the benchmarks should be satisfied to meet this standard:  *For paper-based systems:*   - Number of individuals evaluated for TB disease and TB infection and recorded in the source registers at the health facility matches the number reported (disaggregated by PLHIV, contacts aged <5 years and those aged ≥5 years) - Number of individuals started on TPT in the source register at the health facility matches the number reported (disaggregated by PLHIV, contacts aged <5 years and ≥5 years) - Number of individuals who completed TPT in the source register at the health facility matches the number reported (disaggregated by PLHIV, and household contacts of all ages combined)   *For case-based or patient-based digital datasets:*   - Data validation checks are in place at national level to identify and correct invalid, inconsistent and missing PMTPT data in the minimum set of variables (B4.1) - For each variable in the minimum set (B4.1), ≥90% of individual records are complete, valid and internally consistent for the year being assessed | Met  Partially met  Not met  Not assessed | Met  Partially met  Not met |

BMU: basic management unit; CRVS: civil registration and vital statistics system; HIV: human immunodeficiency virus; PLHIV: people living with human immunodeficiency virus; PMTPT: programme management of TB preventive treatment; TB: tuberculosis; TPT: TB preventive treatment; WHO: World Health Organization.

^a^ This excludes people who are still on treatment at the time of reporting, e.g. those on longer than 12 months treatment regimens.

1. [↑](#endnote-ref-1)
